# Expressive aphasia in a patient with chronic myelomonocytic leukemia

**DOI:** 10.1186/2193-1801-3-406

**Published:** 2014-08-05

**Authors:** Darwin F Yeung, Rose Hsu

**Affiliations:** Department of Medicine, University of British Columbia, 2775 Laurel Street, 10th Floor, Vancouver, BC V5Z 1M9 Canada; Island Medical Program, University of Victoria, Victoria, BC Canada

**Keywords:** Chronic myelomonocytic leukemia, Paraneoplastic syndromes, Autoimmunity, Aphasia

## Abstract

Various paraneoplastic autoimmune phenomena have been reported in patients with myelodysplastic syndromes. We describe a patient who developed expressive aphasia as a paraneoplastic complication of chronic myelomonocytic leukemia (CMML). Awareness of the various possible manifestations of CMML may aid in the early recognition of the condition.

## Background

Chronic myelomonocytic leukemia (CMML) is a clonal hematopoietic stem cell disorder that exhibits both myelodysplastic and myeloproliferative features and is characterized by persistent absolute monocytosis (Parikh and Tefferi 2013). Median survival ranges from 10 to 32 months (Patnaik et al. 2013). Diagnosis may be aided by awareness of its various manifestations, including autoimmune paraneoplastic phenomena (Saif et al. 2002; Enright et al. 1995; Craig and Lin 2013; Fleming et al. 2012; Lemasle et al. 2010; Messiaen et al. 1996). We describe a patient with expressive aphasia as the presenting symptom of CMML.

## Case description

A 64-year-old previously healthy woman presented to hospital in October 2011 with loss of consciousness, expressive aphasia, and left lower extremity numbness. Exam revealed left lower extremity involuntary movements as well as fluent aphasia with paraphasic errors, impaired comprehension, impaired reading, and preserved writing. Computed tomography (CT), magnetic resonance imaging (MRI), and electroencephalography (EEG) were unremarkable. Her symptoms resolved without intervention within days. She began a trial of phenytoin for presumed seizures but discontinued it shortly after discharge due to intolerance.

That admission, she demonstrated microcytic anemia (hemoglobin 8.8 g/dL (range 12.0-15.0), mean corpuscular volume (MCV) 74 fL (range 82–98)), thrombocytopenia (platelet count 107 × 10^9^/L (range 150–400)), normal white blood cell (WBC) count (7.4 × 10^9^/L (range 4.0-10.5)) and monocytosis (1.10 × 10^9^/L (range 0.10-0.80)). Her anemia had been present for over a decade but had not been investigated by a bone marrow biopsy.

Four months later, she re-presented with aphasia, confusion, headache, and left-sided numbness that gradually resolved without intervention. She lost 18 kg since her last admission. CT, MRI, and EEG now showed a subacute left cerebellar infarct but no other abnormalities. Investigations revealed persistent microcytic anemia, thrombocytopenia, and monocytosis, along with neutrophilia (6.99 × 10^9^/L, range 2.00-6.00) and an enlarged spleen (13.5 × 13 × 11 cm). A bone marrow biopsy and cytogenetic analysis demonstrated panhyperplasia and no detectable clonal abnormality. She was discharged on gabapentin for presumed seizures.

Two months later, her aphasia returned with accompanying pericardial effusion, biopsy-proven leptomeningeal inflammation, and left elbow arthritis responsive to prednisone. Rheumatoid factor was transiently elevated at 21.8 IU/mL (range 0.0-20.0). She developed severe leukocytosis (WBC 75.1 × 10^9^/L) from neutrophilia (54.82 × 10^9^/L) and monocytosis (11.26 × 10^9^/L). However, repeat bone marrow biopsy revealed only reactive hyperplasia. Her gapapentin was replaced with valproic acid, methotrexate, and a tapering dose of prednisone.

Over the next 18 months, she had no further recurrences of aphasia but failed to thrive due to an enlarging spleen (25 × 16 × 12 cm). A third bone marrow biopsy in September 2013, after two years of investigation, demonstrated CMML. She started hydroxyurea to reduce the size of her spleen and discontinued prednisone and methotrexate given her neurological remission.

Two months after diagnosis, she was re-admitted for a fourth episode of aphasia that resolved after the reinstitution of prednisone. Given the substantial morbidity from her massive spleen, she underwent a splenectomy. Unfortunately, she developed respiratory failure due in part to leukostasis (WBC 253.4 × 10^9^/L) and died on the second post-operative day.

## Discussion

To our knowledge, this is the first reported case of CMML presenting with expressive aphasia. Its recurrence during disease progression along with its responsiveness to steroids support it being a paraneoplastic autoimmune complication. Previously reported neurologic manifestations of CMML include Guillain-Barre syndrome (del Campo Fernández et al. 2001), IgA-related polyneuropathy (Maeda et al. 1989), and acute and chronic inflammatory demyelinating polyneuropathies (Konstadoulakis et al. 1993; Isoda et al. 2009) peripherally, as well as seizures (Enright et al. 1995), right hemiparesis (Takubo et al. 1998), meningeal inflammation (Aoyama et al. 2003; Ohno et al. 1988), suprachoroidal hemorrhage (Shaikh et al. 2002), pseudotumor inflammatory lesions (Joubert et al. 2013), and anterior ischemic optic neuropathy (De Smit and O’Sullivan 2013) centrally. Awareness of the various manifestations of the condition, including aphasia, however rare, will hopefully alert clinicians of CMML as a diagnostic possibility.

## Consent

Written informed consent was obtained from the patient’s next of kin for the publication of this report.
